# H3K4 Methylation in Aging and Metabolism

**DOI:** 10.3390/epigenomes5020014

**Published:** 2021-06-18

**Authors:** Chia-Ling Hsu, Yi-Chen Lo, Cheng-Fu Kao

**Affiliations:** 1 Institute of Cellular and Organismic Biology, Academia Sinica, Taipei 11529, Taiwan; plusohsu@gate.sinica.edu.tw; 2 Graduate Institute of Food Science and Technology, National Taiwan University, Taipei 10617, Taiwan; loyichen@ntu.edu.tw

**Keywords:** H3K4 methylation, aging, metabolism

## Abstract

During the process of aging, extensive epigenetic alterations are made in response to both exogenous and endogenous stimuli. Here, we summarize the current state of knowledge regarding one such alteration, H3K4 methylation (H3K4me), as it relates to aging in different species. We especially highlight emerging evidence that links this modification with metabolic pathways, which may provide a mechanistic link to explain its role in aging. H3K4me is a widely recognized marker of active transcription, and it appears to play an evolutionarily conserved role in determining organism longevity, though its influence is context specific and requires further clarification. Interestingly, the modulation of H3K4me dynamics may occur as a result of nutritional status, such as methionine restriction. Methionine status appears to influence H3K4me via changes in the level of *S*-adenosyl methionine (SAM, the universal methyl donor) or the regulation of H3K4-modifying enzyme activities. Since methionine restriction is widely known to extend lifespan, the mechanistic link between methionine metabolic flux, the sensing of methionine concentrations and H3K4me status may provide a cogent explanation for several seemingly disparate observations in aging organisms, including age-dependent H3K4me dynamics, gene expression changes, and physiological aberrations. These connections are not yet entirely understood, especially at a molecular level, and will require further elucidation. To conclude, we discuss some potential H3K4me-mediated molecular mechanisms that may link metabolic status to the aging process.

## 1. Introduction

Biological aging is characterized by the gradual accumulation of incidental damage at the cellular, tissue, and organismal levels. This accumulated damage causes homeostatic imbalances that may ultimately lead to a state of disease, such as cardiovascular and neurodegenerative diseases, metabolic disorders and cancer. There is no commonly accepted singular mechanism underlying the aging process, but a combination of factors including genetics, environmental stimuli and metabolic changes have been shown to contribute to the process [1,2,3]. Over the last few decades, the scope of interest in aging research has broadened greatly, especially with regard to the molecular mechanisms underlying the aging process. Now, aging is typically thought of as a complex molecular program, largely because researchers have been able to increase/decrease lifespans of model organisms by numerous genetic and pharmacological means [4,5,6,7,8,9]. Nine tentative cellular and molecular hallmarks of aging have been proposed to act together as determinants of age-related phenotypes; these include genomic instability, telomere attrition, epigenetic alterations, loss of proteostasis, deregulated nutrient-sensing, mitochondrial dysfunction, cellular senescence, stem cell exhaustion, and altered intercellular communication [10]. In particular, the study of epigenetic alterations in aging has recently attracted substantial interest, owing to the fact that epigenetic markers can mediate the physiological effects of environmental stimuli, such as dietary manipulations or stress, acting as a molecular interface for gene–environment interactions throughout the lifespan of an organism [11]. Moreover, an ever increasing number of studies have established links between epigenetic changes and aging or age-related disorders and diseases [12,13].

During the process of aging, many types of epigenetic changes have been shown to occur in response to endogenous and exogenous stimuli. These changes include global changes in DNA methylation and histone modification patterns, the substitution of specialized histone variants for canonical histones, a global loss of core histones or the heterochromatin architecture, and alterations in long non-coding RNA expression [14]. Together, such epigenetic mechanisms influence transcription and can contribute to the major alterations in gene patterns that have been consistently reported in aging cells [15,16,17,18]. Among the diverse modes of age-related genomic regulation, histone modifications are of interest for several reasons. First, multiple histone modifications with specialized epigenetic mechanisms, such as histone 3 lysine 4 tri-methylation (H3K4me3; activating mark) and H3K27me3 (repression mark), may help drive changes in gene expression that have been directly connected to lifespan in many organisms [2]. Second, several histone modifications, particularly methylation/acetylation-mediated histone modifications, are highly responsive to metabolic alterations. Changes in metabolism are the most common sign of aging within cells and thus may serve as an integration platform through which various metabolic signaling may converge to elicit specific age-related cellular responses [19,20]. Third, the potentially reversible nature of histone marks makes it conceivable that one could therapeutically revert the modifications to rescue age-related pathologies [21].

Lysine methylation of unstructured histone tails is an excellent example of a mechanism by which histone modifications intersect with nutrition and cellular metabolism to potentially influence gene expression and aging (or extend lifespan). Histone methylation is affected by metabolism through changes in the concentration of the metabolite, S-adenosylmethionine (SAM), which serves as the methyl donor for SAM-dependent methyltransferases to catalyze the methylation reaction. SAM is mainly derived from methionine, and the manipulation of the methionine levels has proven to be one of only a few dietary interventions that can robustly extend lifespan [22,23,24]. Furthermore, recent works show that the status of key histone marks, such as H3K4me3, can be dynamically tuned by the status of metabolic flux through methionine metabolism and the sensing of methionine bioavailability, which in turn alters gene expression [25,26]. Supporting this notion, other studies have confirmed that the changes in SAM levels or in the levels of certain metabolites that alter the activity of demethylases cause global changes in the patterns of histone methylations [27,28,29,30,31,32,33]. In parallel, numerous lines of evidence have linked changed levels of histone modifications to aging [34,35]. However, whether or not these histone marks can directly function as a metabolic sensor to couple cellular metabolic status with aging remains an open question, as the molecular mechanisms underlying how histone modification changes contribute to aging biology and longevity remain to be further explored. In this review, we focus on the specific epigenetic mark-H3K4 methylation (H3K4me), given its prominent association with gene transcription and its intricate connections with metabolism and aging-associated phenotypes. We first summarize current knowledge regarding the role of H3K4me in regulating aging in different species. Then, we present evidence linking this modification to metabolic pathways. Finally, we discuss potential molecular mechanisms by which H3K4me may function as an interface for extracellular signals to exert genomic effects that influence aging phenotypes.

## 2. H3K4 Methylation Marks Gene Expression across the Genome

While lysine methylation on histones and its potential role in transcription was first described in the 1960s [36,37], it was about a decade later that specific lysine methylation sites on histones including H3K4me were identified in trout testes [38]. In subsequent studies, H3K4me was linked to gene activation in many eukaryotic organisms [39,40]. In general, H3K4me3 deposition peaks at the 5′ end of transcribed genes, close to the transcription start site, and the peak is followed by a downstream gradient of H3K4 di-methylation (H3K4me2) and H3K4 mono-methylation (H3K4me1) deposits in the gene bodies of longer transcription units. In recent years, with advances of the ChIP-seq (chromatin immunoprecipitation followed by sequencing) and MS (mass spectrophotometry)-related methods, the genome-wide distributions and abundances for all three states of H3K4me have been intensively assessed. In human cells, the global abundance of H3K4me3 comprises only about 1% of all H3K4me states. On the other hand, H3K4me2 and H3K4me1 are broadly distributed, with respective global abundances estimated at 1–4% and 5–20%, respectively [41]. H3K4me3 not only defines active transcription initiation sites at most promoters, but it is also functionally linked to other nuclear events and various cellular processes [42,43,44,45,46,47]. Similarly, H3K4me2 shows specific association with transcription factor binding regions [48] and tissue-specific intragenic *cis*-regulatory elements [49], while H3K4me1 is thought to mark enhancers [50,51]. However, studies in yeast and mammals show that loss of H3K4me only induces minor changes in gene expression [52,53,54], and some evidence has even suggested that H3K4me may passively accumulate on genes as a result of transcription, rather than exerting active functions [55]. Therefore, whether and how H3K4me contributes to the regulation of gene expression remains to be further clarified. Interestingly, recent studies in mammals suggest that atypical broad H3K4me3 domains are important for transcriptional precision at key cell identity/function genes [56] and for cell-type-specific gene expression [57,58].

The involvement of H3K4me3 in regulating gene expression across the lifetime of a cell has also been investigated recently in budding yeast (*Saccharomyces cerevisiae*) and worms (*Caenorhabditis elegans*) [59,60]. The studies in yeast have revealed impairments in the upregulation of aging-linked genes in H3K4me3-defective cells, coincident with reduced lifespan. In yeast cells, H3K4me3 normally has both repressive and inducing activities with regard to gene expression [40,52,61], but the inducing activity appears to be more important for a large subset of genes to attain normal expression levels, and the repressive capacity of H3K4me appears to decline as cells age. These studies provided an important demonstration that H3K4me3 is an active epigenetic mark that facilitates gene expression, and this mark is critical for maintaining cell viability throughout the lifespan of yeast [59]. Similarly, H3K4me3 in the somatic cells of *C. elegans* was reported to affect the expression of a set of genes with functions that are commonly implicated in aging biology. The genes marked by H3K4me at adult stages show age-dependent, correlated changes in H3K4me3 patterns and RNA expression [60]. Thus, further mechanistic investigations will be required to better understand how age-dependent changes in H3K4me3 help to regulate chromatin states and transcription during aging in these and other organisms.

## 3. H3K4 Methylation Is Involved in Biological Functions Other than Transcription

**Figure 1:**
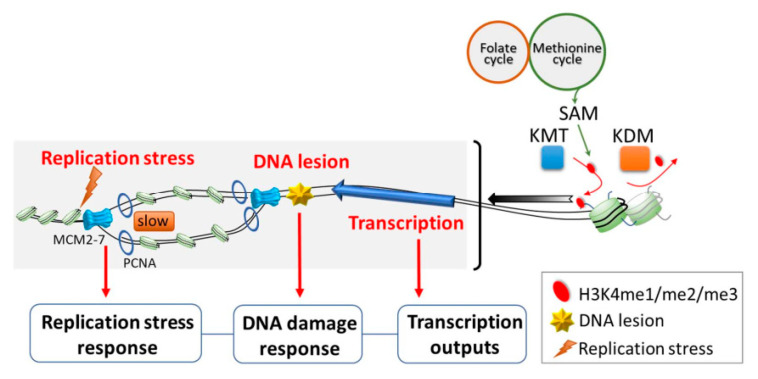
H3K4 methylation is associated with various biological functions. H3K4me is involved in a variety of mechanisms affecting nuclear functions. The epigenetic mark is thought to promote gene transcription, and there is an increasing body of evidence showing that H3K4me is also involved in various other nuclear functions, such as origin firing, replication stress, and DNA damage responses.

Based on the studies mentioned above, H3K4me is thought to largely promote gene transcription. However, there is an increasing body of evidence showing that H3K4me is also involved in various other biological functions (Figure 1). For example, a positive role of H3K4me in regulating origin firing has been suggested, as it is enriched near human early-firing origins relative to late-firing origins [62,63,64]. Origin firing is the initiation of DNA replication, and it takes place at well-defined replication origins in budding yeast [65]. In metazoans, origin firing occurs at specialized start sites thought to be determined by a combination of DNA sequence and chromatin-associated factors [66]. At these sites, DNA is first unwound by loading of pre-replicative complexes (pre-RCs) [67]. Then, replisomes are assembled and activated under the regulation of two kinases, S-CDK (S-phase cyclin-dependent kinase; Cdc28 complexed with either Clb5 or Clb6) and DDK (Dbf4-dependent kinase; Cdc7 complexed with Dbf4) [68]. It is known that not all replication origins are fired at the same time. While the molecular mechanisms underlying the preferential distribution of CDK and DDK to specific origins are not well understood, the kinetochore protein, Ctf19, and the forkhead proteins, Fkh1 and Fkh2, are thought to be involved [69,70,71]. Nevertheless, the DNA sequence and chromatin context at an origin appear to have the greatest influence on its likelihood of undergoing initiation [72,73,74].

In addition to the role of H3K4me in human cells noted above, genome-wide profiling of H3K4me3 in synchronized yeast cells revealed that the mark is more readily erased during S-phase at early origins than late origins [75]. The enrichment of H3K4me3 at early-firing origins (among a subset of defined origins) has also been demonstrated in synchronized human and monkey cells [76]. Remarkably, Rizzardi et al. provided genetic evidence that H3K4me has functional consequences on origin activities. As such, they found that two enzymes required for the methylation of H3K4 are necessary for robust growth of several hypomorphic replication mutants [47]. Similarly, the loss of mammalian H3K4 histone demethylase was shown to exert adverse effects on the initiation of early origins, suggesting the direct involvement of H3K4me in this process [77]. Thus, current knowledge supports the idea that the methylation status of H3K4 is regulated by a concerted mechanism and affects origin activity.

Some other observations further imply that H3K4me may be crucial to protect cells from replication perturbations. For example, H3K4me protects stalled replication forks from deleterious degradation, thus playing a role in maintaining genome stability [78]. Moreover, H3K4me functions downstream of yeast Rad53 in the S-phase checkpoint to prevent transcription–replication conflicts (TRCs), which represent a major source of genome instability [45]. Interestingly, H3K4me also appears to be important for the efficient repair of DNA double-strand breaks, as the loss of H3K4me impairs the non-homologous end joining pathway and slows progression through S-phase in the presence of replication stress [44]. Together, these results indicate that H3K4me is important for faithful DNA replication under both normal growth conditions and replication stress.

In addition to gene regulation and replication, H3K4me also regulates other cellular processes, such as mitosis and apoptosis. H3K4me was shown to play a role in regulating the response to benomyl, a microtubule-destabilizing agent. Notably, two different mechanistic explanations for the benomyl-resistant phenotype of H3K4me-deficient cells were provided in two separate papers. Schibler et al. described an intriguing molecular mechanism wherein H3K4me3 regulates the spindle assembly checkpoint (SAC) by directly binding to the activated form of Mad2, a key component of SAC, thus limiting its availability [42]. Interestingly, an alternative mechanism was proposed by another study. The authors of that study found that Set1-H3K4 methylation is required for proper cell-cycle-dependent gene expression. A lack of Set1 delays entry into S-phase, which somehow also impacts the metaphase–anaphase transition. Thus, they proposed that H3K4 methylation regulates the cross-talk between G1-S transition and mitosis [43]. While more work will be required to unravel this apparent discrepancy, the studies agree on the basic principle that H3K4me is important in coordinating cell cycle progression and the proper assembly of the mitotic spindle during mitosis [42,43]. On the other hand, the loss of H3K4me has been identified as a novel trigger for apoptosis in yeast; this study will be further discussed in a later section [46]. Despite our lack of understanding about the nuanced role of H3K4me in different cellular contexts, mounting evidence overwhelmingly supports the idea that H3K4me plays a multifunctional and context-dependent role in many essential cellular processes.

## 4. Writing and Erasing H3K4 Methylation

**Figure 2:**
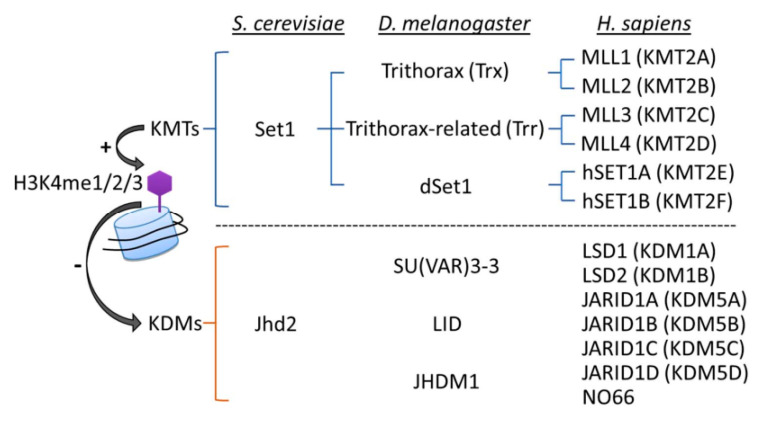
List of histone lysine methyltransferases (KMTs) and demethylases (KDMs) responsible for H3K4 methylation in various species. Blue lines indicate the phylogenetic relationships between H3K4 KMTs from yeast (*S. cerevisiae*), fruit flies (*D. melanogaster*) and humans (*H. sapiens*).

The state of H3K4me (mono-(me1), di-(me2), or tri-methylation (me3)) is reversibly regulated by the actions of histone lysine methyltransferases (KMTs; “writers”) and lysine demethylases (KDMs; “erasers”) (Figure 2). It is clear that H3K4me is a highly dynamic process, with the loss of basal activity of KMTs or KDMs, respectively, causing genome-wide decreases or increases in H3K4me levels. The structures and activities of KMTs and KDMs have been extensively reviewed elsewhere [79,80,81]; however, some relevant details will be mentioned here.

H3K4 methylation is catalyzed by a series of KMTs, using SAM as a methyl group donor. In *S. cerevisiae* (yeast), Set1 is the sole H3K4 KMT. The yeast complex associated with Set1 (COMPASS) consists of a catalytic subunit Set1 and the concerted action of seven regulatory subunits (Swd1, Swd2, Swd3, Bre2, Sdc1, Spp1 and Shg1), which are responsible for generating H3K4me1, H3K4me2, and H3K4me3 [79]. *Drosophila melanogaster* (fruit fly) carries three Set1 homologs, dSet1, Trithorax (Trx), and Trithorax-related (Trr); dSet1 is responsible for depositing the bulk of H3K4me2 and H3K4me3 [82,83]. In humans, there are six Set1-related proteins that form MLL/COMPASS complexes, including two homologs of Set1—hSET1A and hSET1B—and the mixed-lineage leukemia (MLL) family (MLL1, 2, 3 and 4). MLL1 and MLL2 are homologs of *D. melanogaster* Trx, while MLL3 and MLL4 are homologs of *D. melanogaster* Trr [79]. All of the MLL/COMPASS complexes share a four subunit subcomplex consisting of Wdr5, Rbbp5, Ash2 and Dpy30, which are respective homologs of yeast Swd3, Swd1, Bre2 and Sdc1, and are together known as WRAD [79,84]. The WRAD subcomplex must associate with different KMTs to form various catalytic complexes, and its presence is essential for full enzymatic activity [85,86]. In *C. elegans*, the MLL/COMPASS complex contains a WRAD subcomplex associated with one of two KMTs, either the Set1-like SET-2 or the MLL-like SET-16 [85,87,88]. Of note, each complex is endowed with additional proteins that determine H3K4me status and biological functions. In yeast COMPASS, the WD40 protein Swd2, PHD finger protein Spp1, and Shg1 may be present [79]. In mammalian cells, unique subunits, such as WDR82 (WD repeat domain 82) in the hSET1A complex, menin in MLL1/2, and PTIP (Pax transactivation domain-interacting protein) in MLL3/4, have roles in regulating H3K4me and recruiting distinct transcription factors for the expression of specific target genes [89,90,91].

Histone methylation was originally thought to be stable and irreversible [92]. However, in 2004, the discovery of the first H3K4 KDM, human lysine-specific demethylase 1A (KDM1A; also known as LSD1), revealed that H3K4me2 and H3K4me1 can both be removed by KDM1A/LSD1 through an FAD (flavin adenine dinucleotide)-dependent oxidation reaction; thus, histone methylation was shown to be reversible and dynamically regulated [93]. After that pioneering study, JARID1 (jumonji AT-rich interactive domain-1) family proteins (JARID1A/KDM5A, JARID1B/KDM5B, JARID1C/KDM5C and JARID1D/KDM5D) and the JmjC domain-containing protein (nucleolar protein 66 (NO66); also known as MAPJD) were found to serve as H3K4 demethylases in human cells [94,95]. It is noteworthy that unlike KDM1A/LSD1, which catalyzes demethylation specifically on H3K4me1/me2, the KDM5A-D proteins can function as demethylases for all three states of H3K4me [94,96].

Much of the evidence supporting the idea that H3K4me may play a crucial role in aging comes from studies in which the levels of KMTs and KDMs were modulated in *C. elegans*. In the *C. elegans* genome, there are three KDM1 genes (*amx-1*, *spr-5*, and *lsd-1*) and one KDM5 gene (*rbr-2*). The *amx-1* gene encodes the sole KDM1B enzyme, while the other two KDM1 genes are KDM1A homologs. The sole KDM5 member, RBR-2, can act as a demethylase for H3K4me3 and H3K4me2, but it is more efficient at removing H3K4me3 [97]. In contrast, the KDM1 members (SPR-5, LSD-1, and AMX-1) are responsible for removing H3K4me1 and H3K4me2 [98,99,100,101,102]. Interestingly, targeting different classes of KDMs (KDM1 versus KDM5) may differentially affect the aging process [103,104]. Despite this context specificity, it is clear that KDM-modulated H3K4 demethylation is functionally relevant during aging, and follow-up studies to delineate the mechanisms will help reconcile any apparent discrepancies [105,106,107].

## 5. Roles of H3K4 Methylation in Regulating Aging in Different Species

### 5.1. H3K4 Methylation Contributes to Pathways That Regulate Yeast Aging and Lifespan

Like *C. elegans*, the budding yeast, *S. cerevisiae*, is widely recognized as one of the simplest and most powerful organisms for aging research. Yet, despite the simplicity of this unicellular eukaryote, the mechanisms underlying yeast-aging phenotypes are surprisingly similar to those seen in human post-mitotic cells [108]. Aging in yeast cells can be modeled chronologically and is assessed by monitoring cell survival in stationary batch cultures after the diauxic shift. Thus, the definition of chronological lifespan (CLS) is the length of time that yeast survive in the stationary phase following nutrient scarcity. In contrast to the CLS model of aging in post-mitotic (or differentiated) cells, replicative lifespan (RLS) represents an aging model for proliferating cells (e.g., undifferentiated stem cells) and is defined as the number of times a mother cell divides and produces a daughter cell before it enters senescence and eventually dies [109].

In yeast, a hint that H3K4me may play a role in aging first came from the observation that cells with loss of H3K4me, due to deficiencies in any of the three subunits of COMPASS (Set1, Spp1, and Bre2), exhibit a shortened CLS [46]. However, the link between H3K4me and the aging process was still not clear, as Walter at al. further showed that the loss of H3K4me during chronological aging can trigger cell death in yeast, which could explain the shorter lifespan [46]. Recently, the importance of H3K4me3 in regulating dynamic transcriptional changes in response to aging has been demonstrated [59]. A link was made between H3K4me3 and the inducible expression of a subset of genes known to be upregulated with age, highlighting the direct role of H3K4me3 in maintaining the normal expression of many genes across the lifespan. Consistent with this notion, age-associated H3K4me3 dynamics in *C. elegans* were shown to be highly correlated with changes in RNA expression of genes commonly implicated in aging biology [60].

### 5.2. H3K4 Methylation Modulates Aging in Nematodes and Fruit Flies

H3K4me deposition has strong implications for aging not only in yeast but also in nematodes and fruit flies. In *C. elegans*, RNAi knockdown or mutations in genes encoding members of the H3K4 KMT complex (*ash-2*, *set-2* and *wdr-5*) lead to H3K4me3 deficiency and concomitantly extend lifespan [103]. Moreover, the lifespan extension phenotype caused by ASH-2 knockdown is suppressed by the sole *C. elegans* KDM5 demethylase, RBR-2, suggesting an epistatic relationship between these two opposed H3K4me modifiers [103]. However, the effects of reducing RBR-2 function on lifespan remain unclear. While some studies, including Greer et al., showed that RBR-2-mediated H3K4 demethylation may promote aging [103,104], others have suggested that RBR-2 deficiency, which presumably upregulates levels of H3K4me3, leads to increased lifespan [100,110,111]. These discrepancies might be explained by the effects of some unidentified H3K4 methyltransferases (other than H3K4 KMTs), which could be influenced by RBR-2 to affect a different set of downstream target genes. Nevertheless, reduced function of KMD1A/LSD-1, another H3K4me KDM in *C. elegans*, has been reported to increase lifespan [104,112].

In *D. melanogaster*, a deficiency in Lid, the homolog of RBR-2, causes a decrease in the lifespan of male flies, although the same effect on lifespan was not observed in female flies [113]. Intriguingly, effects on lifespan are not always observed when modulating H3K4me regulators in flies; for example, loss-of-function in Trr, a member of another H3K4 KMT complex in *D. melanogaster*, does not alone affect the lifespan of male flies [114].

### 5.3. H3K4 Methylation Is Associated with Age-Related Diseases in Mouse Models

A study to profile transcriptome, DNA methylome, and histone modifications of young and old murine hematopoietic stem cells (HSCs) revealed an age-dependent increase in the breadth of more than 50% of all H3K4me3 peaks (especially on genes in which H3K4me3 is already broad), which most likely contributes to the dysfunction of HSCs in the older animals [115]. Furthermore, the broadened peaks tend to be associated with genes that determine HSC identity [115], suggesting that the increase in this activating mark on these particular genes may be linked to functional changes in aged HSCs. This argument is also consistent with the findings of another study that showed the broadest H3K4me3 domains control the cell-identity-related genes by increasing their transcriptional consistency [56]. On the other hand, a recent study using a similar integrative characterization of epigenomic and transcriptomic changes during normal human HSC aging demonstrated that aging is associated with significant reductions in H3K27ac (activating mark), H3K4me1 and H3K4me3; furthermore, the sites with decreased H3K4me1 were associated with genes involved in myeloid and erythroid differentiation and function, while those with lost H3K27ac were linked to genes involved in leukocyte activation, apoptotic signaling and histone modifications [116]. More importantly, the molecular pathways targeted by this age-related epigenetic reprogramming were shown to be similarly altered in acute myeloid leukemia (AML), suggesting that such reprogramming with age may predispose individuals to the development of AML [116].

The role of epigenetics in age-related disease was also investigated in an Alzheimer’s disease (AD) mouse model [117]. In the AD mice, H3K4me3 and its catalyzing enzymes are significantly increased in the prefrontal cortex (PFC), a key brain region impaired in AD, and treating AD mice with a specific inhibitor of H3K4 KMTs results in substantial recovery of PFC functions [117]. This study not only revealed a novel role for H3K4me3 in AD pathophysiology, but it also predicts a novel molecular target for AD therapy, providing a potentially valuable resource for further studies on AD and age-related neurodegenerative disorders.

Recently, Sleiman et al. used multiple genomic methods to measure changes in the transcriptome, DNA methylome and histone modifications in three metabolic tissues (liver, heart and muscle) of adult and aged mice [118]. Among the three tissues, heart and muscle show global increases in chromatin accessibility, and the Gene Ontology (GO) enrichments for genes with both increased H3K27ac (activating mark) and decreased H3K27me3 (repression mark) imply a role for open chromatin in aging. However, no functional enrichments for changes in H3K4me3 were observed in the three tissues [118]. As for histone modifications, the results indicate that the epigenetic footprint of aging is strikingly tissue specific, with the greatest changes of H3K4me3 observed in muscle [118]. An age-related decline in muscle mass and function is called sarcopenia and considered to be a major risk factor for disease in the elderly [119]. In human aging cohort studies, sarcopenia was suggested to have origins in fetal and early life [120,121], suggesting that epigenetic modifications, such as H3K4 methylation, are likely to underpin muscle memory [122].

### 5.4. The Relationship between Regulation of H3K4 Methylation and Cell Senescence In Humans

Cellular senescence is a state of stable cell cycle arrest that has been implicated in aging, as the accumulation of senescent cells is associated with aging and age-related diseases [123,124]. In human cells, a profound redistribution of H3K4me3 in the genome occurs upon senescence [125]. In their study, Shah et al. compared genome-wide H3K4me3 distributions in primary skin fibroblasts of controls and HGPS (Hutchinson–Gilford progeria syndrome) patients, who have a segmental premature aging syndrome associated with accelerated cell senescence. In this comparison, the authors found large-scale chromatin changes, including the acquisition of large contiguous stretches of altered H3K4me in HGPS patients. Gene expression analysis further showed that H3K4me3 is enriched at upregulated genes, and it is depleted at downregulated genes, especially those with functions in cell cycle and proliferation. These results revealed a surprising link between global changes in H3K4me3 distribution and accelerated aging, presumably due to chromatin changes during premature senescence [125]. However, the functional implications of large-scale epigenomic alterations in senescent cells are not yet clear. As broad H3K4me domains are important for the precise regulation of transcriptional activity for key cell identity/function genes [56], it is possible that the broadly spread H3K4me3 deposits observed during senescence/aging could influence the expression of particular gene sets to affect the cellular phenotype. An alternative possibility is that the profound change in H3K4 methylation distribution may contribute to genome reorganization. Deficiency of Cfp1, a conserved subunit of the Set1 complex in mice, causes a shift of H3K4me3 from the promoters of expressed genes to numerous “ectopic sites”; however, there are minimal consequences for transcription. Further analysis revealed that these ectopic peaks are enriched in cohesion- and CTCF-binding sites, which are thought to mediate chromatin looping [126]. Moreover, chromosome folding analysis in budding yeast by a Hi-C-based method, called Micro-C, revealed abundant chromosome interaction domains (CIDs), which are similar to the reported topologically associating domains (TADs) in mammals [127]. Strong boundaries between CIDs occur at promoters of highly transcribed genes, and intriguingly, nucleosomes at the boundaries exhibit significant enrichments of a variety of histone marks at the 5′ ends of genes [128], including high levels of H3K4me3. Together, these results imply a role for H3K4 methylation in chromatin organization. Furthermore, a global genetic analysis of gene pairs in yeast revealed that the deletion of *SET1* positively interacts with mutations in subunits of cohesin and condensin [129], which suggests functionally proximal relationships between the proteins. Thus, there is good reason to expect a possible functional role for H3K4me in regional and/or global chromatin organization.

H3K4me3 also exists alongside the repressive H3K27me3 mark in so-called bivalent chromatin domains, which are usually seen at promoters of key developmental regulators in stem or progenitor cells. In stem cells, the genes marked with bivalent domains are poised to be quickly activated upon the loss of H3K27me3 during differentiation and development. Intriguingly, the bivalent chromatin domain promoters are preferential sites for DNA hypermethylation in some aged human tissues, and the sites are also methylated and silenced in cancers [130,131]. Given the nature of their pro-differentiation functions, bivalent-marked genes tend to have tumor suppressor-like properties, meaning that their methylation and stable silencing can promote proliferation, self-renewal and malignancy. These studies support the idea that age-associated methylation changes in bivalent chromatin domains might act as one of the multiple “hits” required for the transformation of aged cells.

## 6. Metabolic Signaling Pathways Involving H3K4 Methylation

### 6.1. One-Carbon Metabolism

**Figure 3:**
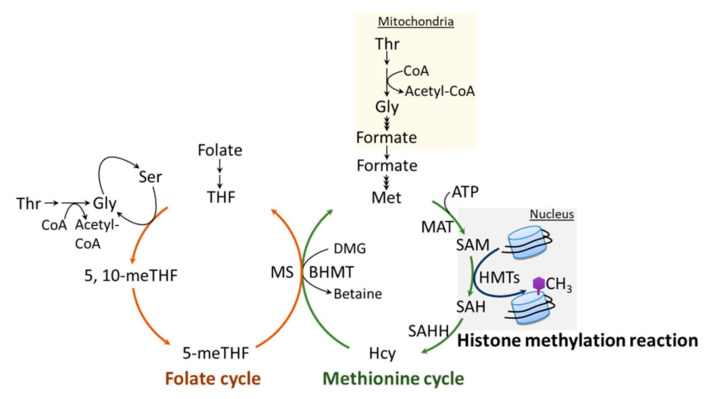
Histone methylation reaction and one-carbon metabolism. *S*-adenosylmethionine (SAM) is synthesized from ATP and methionine (Met) by methionine adenosyltransferase (MAT). Histone methyltransferases (HMTs), such as H3K4 KMTs, utilize SAM as a cofactor to transfer a methyl group to the histone substrate, yielding *S*-adenosylhomocysteine (SAH) and a methylated histone. In the methionine cycle, SAH is converted to homocysteine (Hcy) via *S*-adenosylhomocysteine hydrolase (SAHH), followed by the re-methylation of Hcy back to methionine via either methionine synthase (MS) utilizing a methyl group from 5-methyltetrahydrafolate (5-meTHF) or by betaine-homocysteine *S*-methyltransferase (BHMT) utilizing a methyl group from betaine. In the folate cycle, folic acid is reduced to tetrahydrofolate (THF), which can then accept a one-carbon unit from serine (Ser) or glycine (Gly), producing 5-meTHF. Threonine (Thr) is also involved in modulating SAM concentrations, as it provides both glycine and acetyl-CoA required for optimal synthesis of SAM.

The methylation of H3K4, like the methylation of other sites on histones and DNA, requires the metabolite, SAM (universal methyl group donor). SAM is generated by one-carbon metabolism (Figure 3), a fundamental metabolic pathway that couples the folate and methionine cycles. The latter cycle is particularly relevant to histone methylation, as it provides a direct link to histone substrates through the generation of SAM. SAM is synthesized from ATP and methionine by the enzymatic action of methionine adenosyltransferase (MAT), also known as SAM synthetase (SAM-S) [132]. Histone methyltransferases (HMTs), such as H3K4 KMTs, transfer a methyl group from SAM to histone substrates, yielding *S*-adenosylhomocysteine (SAH) and a methylated histone [25,133]. SAH is a potent product inhibitor of SAM-dependent methylation, and it is efficiently converted to homocysteine (Hcy) and adenine via *S*-adenosylhomocysteine hydrolase (SAHH) [134]. The inhibition of SAHH causes abnormal accumulation of SAH, which inhibits the methylation reaction via a feedback mechanism. Cells generally maintain low concentrations of SAH, and the ratio of cellular SAH/SAM is frequently used as an indicator of cellular methylation status. To complete the methionine cycle, Hcy can be remethylated back to methionine by the donation of a methyl group either from 5-methyltetrahydrafolate (5-meTHF) or from betaine, which is catalyzed by methionine synthase (MS) or betaine-homocysteine *S*-methyltransferase (BHMT), respectively.

In the folate cycle, folic acid is reduced to tetrahydrofolate (THF), which can then accept a one-carbon unit from either serine or glycine to generate 5-meTHF. As mentioned above, 5-meTHF can supply a methyl group for the generation of methionine via MS, yielding THF. Notably, metabolites of folate, THF and 5-meTHF are precursors of pyrimidines and purines, which are essential components of nucleic acids.

### 6.2. One-Carbon Metabolism and Its Regulation of H3K4 Methylation

The availability of methionine and other components of the methionine cycle, such as SAM or SAH, can directly influence histone methylation, including the deposition of H3K4me. While earlier evidence provided links between the metabolic regulation of SAM and SAH and the epigenetic status of cells [135,136,137,138,139], the most direct evidence connecting one-carbon metabolism with H3K4me3 comes from a study performed by Mentch et al. In their study, the authors specifically show that in cells and mice, SAM levels and the SAM/SAH ratio, which can be quantitatively tuned through the modulation of methionine bioavailability, are sufficient to drive rapid changes in H3K4me3 levels, leading to altered gene transcription [25]. In addition, this metabolic–epigenetic axis can run in reverse through a signal transduction-mediated feedback mechanism. As such, methionine-restriction-induced decreases in H3K4me3 directly contribute to the transcriptional downregulation of a network of genes involved in one-carbon metabolism [25]. This mechanism might serve to maintain physiological homeostasis. Later, a follow-up study by Dai et al. revealed a specific mechanism of how alterations in methionine metabolism might influence H3K4me3 and change gene expression. In that study, the authors concluded that the peak width is the most informative parameter in H3K4me3 dynamics upon changes in nutrient availability and is indeed linked to the functional outcomes [140]. The observation that low methionine has direct impacts on both SAM and H3K4me3 was also made in mammalian cells (immortalized mouse embryonic fibroblasts) and *S. pombe* [141,142].

Threonine (Thr) is another amino acid that can affect SAM concentrations, as it provides a source of both glycine and acetyl-CoA, which are required for optimal synthesis of SAM. Interestingly, just like the effect of methionine restriction on H3K4me3, either Thr restriction or the depletion of threonine dehydrogenase (a mitochondrial enzyme that hydrolyzes Thr into acetyl-CoA and glycine) in mouse embryonic stem cells (mESCs) could significantly reduce SAM levels and selectively diminish H3K4me2/me3. The disappearance of H3K4me2/me3 was then linked to slowed growth and increased differentiation [138].

As mentioned in the previous section, folate is an essential cofactor in the generation of endogenous methionine. Therefore, several classical studies reported links between folate deficiency and reduced SAM concentrations in different animal and cell models [143,144,145,146]. However, this correlation is not always observed. Other more recent studies have shown that a severe folate deficiency may actually increase SAM concentrations [147,148]. These reported increases in SAM level are likely attributable to SAH-mediated inhibition of methyltransferases. The results also support the idea that SAH concentration may be a sensitive biomarker of cellular methylation status [137,143,149]. Interestingly, in *S. cerevisiae*, the analysis of a folate-auxotrophic (*fol3∆*) revealed that low folate concentrations cause a decrease in H3K4me3/me3 and affect gene expression. Similarly, the SAM levels in *fol3∆* cells do not show significant changes, suggesting a SAM (or even a methionine)-independent regulatory mechanism underlying this metabolic–epigenetic crosstalk. On the other hand, folate has been suggested to play an active and direct role in H3K4me-mediated gene expression. In 2011, the LSD1 H3K4 demethylase was identified as a folate-binding protein, and the binding of folate did not inhibit LSD1 activity [150]. Subsequent studies of the crystal structure of LSD1•CoREST (co-repressor for repressor element 1 silencing transcription factor) complexed with THF (tetrahydrofolate) revealed that the THF molecule is in close proximity to the substrate-binding cavity, occupying the place of the H3K4 side chain. Therefore, the authors concluded that the biological importance of the bound folate is to serve as an acceptor for the byproduct, formaldehyde, thus preventing the accumulation of this potentially toxic molecule [151]. Furthermore, the interplay between folate, LSD1 and H3K4me was demonstrated in the livers of mice fed with a folate-deficient diet. In the liver, H3K4me is substantially elevated due to decreased activity of LSD1, which results from the lack of available folate to scavenge formaldehyde molecules [152].

In summary, SAM, Thr and folate all have the potential to serve as components of a bio-sensing metabolic mechanism to convey information about metabolic changes to histone modifications, eventually affecting gene expression to drive cellular responses to the overall metabolic state.

### 6.3. Methionine and Its Involvement in Aging

Methionine restriction (MR) is a widely known dietary manipulation that can induce lifespan extension in many different eukaryotic species, ranging from yeast to nematodes, fruit flies and mice [22,23,153,154]. Aside from increasing longevity, other metabolic benefits of MR have also been reported, including decreased inflammation [155,156], reduced oxidative stress [157,158,159], lower body weights [160,161], and increased insulin sensitivity [162,163]. The mechanisms underlying lifespan extension by MR are complicated and still not fully understood. In terms of cell physiology, the activation of autophagic flux and concomitant vacuole acidification have been suggested as potential mechanisms in yeast, as the deletion of autophagy-related genes or lowering vacuolar acidification abrogates MR-mediated CLS extension [153]. In addition, MR-mediated CLS extension at least partially relies on the key signaling protein of the mitochondrial retrograde response (mitochondrial–nuclear communication), as loss-of-function in Rtg3 suppresses the changes in 20% of differentially expressed genes under MR [164].

However, the molecular mechanisms underlying the MR-mediated lifespan extension remain to be elucidated. The epigenetic response to MR has been examined in recent work, as it relates to aging [14]. The ratio of SAM/SAH in cell culture media, which can be directly altered by methionine concentrations in a physiologically relevant range, may affect the methylation state of H3K4 and gene regulation [25]. Moreover, upon MR, the methionine concentrations in human serum are affected enough to change histone methylation [25]. Similarly, MR-induced alterations in DNA methylation and concomitant decreases in SAH were observed only in adult but not young mice fed with low-methionine diets, in an age-dependent effect [165]. Taking these data together, it is tempting to speculate that MR-induced changes in SAM and/or SAH availability might be capable of altering epigenetic profiles that might contribute to lifespan regulation. However, so far, no direct linkage between MR-induced epigenetic changes and aging phenotypes has been made, even though previous studies collectively support the idea that MR causes alterations in epigenetic marks. In addition, given the complexity of the aging process, it is a near certainty that multiple mechanisms are involved in mediating MR effects on lifespan. For example, a decrease in SAM levels upon MR was shown to promote lifespan extension via SAMTOR-dependent inhibition of the mTORC1 (mechanistic target of rapamycin complex 1) signaling pathway [166]. This complexity may impede the interpretation of experimental results, as some mechanisms may be masked or especially important under certain conditions.

## 7. Conclusions and Perspectives

In this review, we summarize current knowledge regarding the role of H3K4me in the aging of eukaryotes. Overall, the molecular mechanisms of how H3K4me contributes to longevity are largely unknown. While alterations in the H3K4me status are not likely to account for many cellular aspects of aging, it is reasonable to expect that H3K4me-mediated alterations in the expression of certain genes are an important determinant of lifespan, as H3K4me has long been strongly associated with transcriptional activation. Indeed, numerous studies have addressed the role of H3K4me in regulating transcription during aging, asking whether the disruption of this process leads to the loss of transcriptional precision that is detrimental to longevity [59,60]. This line of research reflects two common themes in aging studies. First, aging is associated with progressive changes in chromatin marks, and second, age-associated changes in transcription regulatory networks have the potential to impact cellular function, thus contributing to aging phenotypes and diseases [14,167].

**Figure 4:**
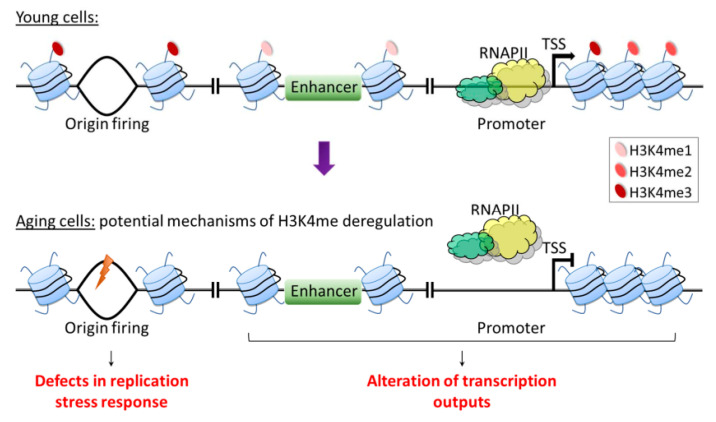
Possible molecular mechanisms of aging-associated H3K4 methylation. A simplified illustration of H3K4me occupancy is presented. Transcription may be modulated by H3K4me3 near promoters, H3K4me2 just downstream of promoters, and promoter-distal H3K4me1. H3K4me3 is also enriched near early-firing origins, and it is involved in faithful DNA replication under both normal growth conditions and replication stress. As patterns of H3K4me change during aging, the functional roles might not only be involved in gene regulation but also in other cellular processes, such as replication stress response. Therefore, future mechanistic studies on the relationships between H3K4me and aging-related processes may provide valuable insights into the underlying mechanisms of aging.

H3K4me deposition can be influenced by nutrition and metabolites involved in methionine metabolism. For example, methionine is utilized in the methionine cycle as a methyl donor in the production of SAM. SAM is then used as a cofactor in the methylation of H3K4, and its deficiency was found to specifically reduce H3K4me at the promoters of key genes involved in cell proliferation of CD4^+^ T helper (Th) cells [168]. Interestingly, MR has long been known to robustly extend lifespan across different species [19]. Based on the nutrition–genome interaction observed in Th cells and the known role of H3K4me in aging, it is tempting to speculate that in the context of aging, H3K4me may act as an interface connecting metabolic signals with the aging process via its function in regulating gene expression. On the other hand, the loss of H3K4me3 only has minor effects on transcription in yeast and mammals [52,54], and H3K4me has been suggested to be a consequence, not a cause of transcription [55]. Thus, it is still debatable whether a role exists for H3K4me in regulating age-associated gene expression. Furthermore, in the study suggesting a mechanistic link between age-dependent H3K4me3 dynamics, gene expression changes, and physiological aberrations in *C. elegans* somatic cells, the conclusions are drawn from changes in 30% of H3K4me3-enriched regions that largely mark gene bodies [60]. This location is incongruent with the canonical model that gene regulatory function may be attributed to H3K4me3 deposited near the promoter region. Thus, it is clear that unique patterns of H3K4me3 are prone to change during aging, but the functional roles might not be solely limited to gene regulation. H3K4me is also known to be involved in various cellular functions, such as DNA replication, replication stress, DNA damage response, the assembly of the mitotic spindle, and apoptosis [42,43,44,45,46,47]. Importantly, impairments of cellular responses to replication stress and DNA damage have been associated with aging [169,170,171], suggesting that these mechanisms also can potentially be influenced by age-dependent H3K4me dynamics (Figure 4). Thus, future studies to investigate and refine the role of H3K4me dynamics in longevity are expected to yield valuable insights into the nature and control of organismal aging.
